# Supplementary material for: A molecular switch regulating transcriptional repression and activation of PPARγ
Source: Nat Commun. 2020 Feb 19;11:956. doi: 10.1038/s41467-020-14750-x (PMC7031403; doi:10.1038/s41467-020-14750-x)
Supplement: Supplementary file 1 — Supplementary Information [file 41467_2020_14750_MOESM1_ESM.pdf]

Supplementary Information

A Molecular Switch Regulating Transcriptional Repression and Activation of PPARγ

J. Shang *et al.*

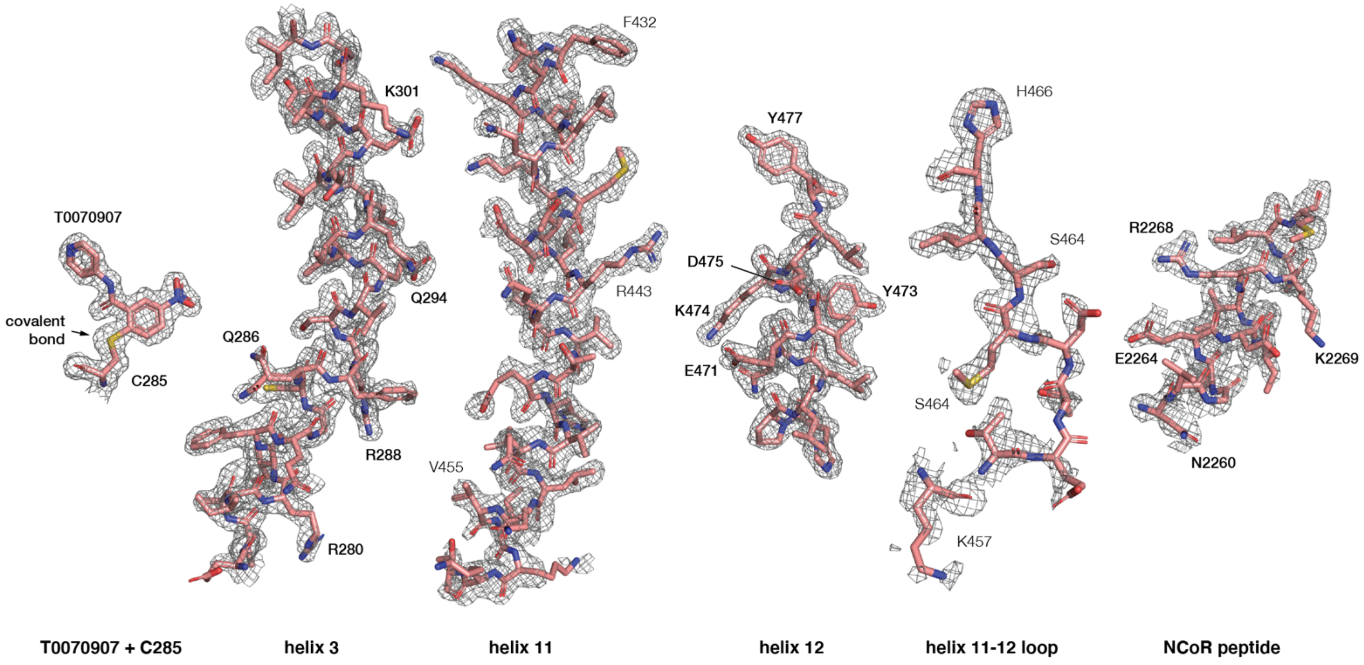

Supplementary Fig. 1. Omit map ($2F_O–F_C$, contoured at 1σ except the helix 11-12 loop is contoured at 0.8 σ) for the ligand, key secondary structural elements, and peptide in the crystal structure of PPARγ LBD bound to T0070907 and NCoR ID2 peptide (PDB 6ONI).

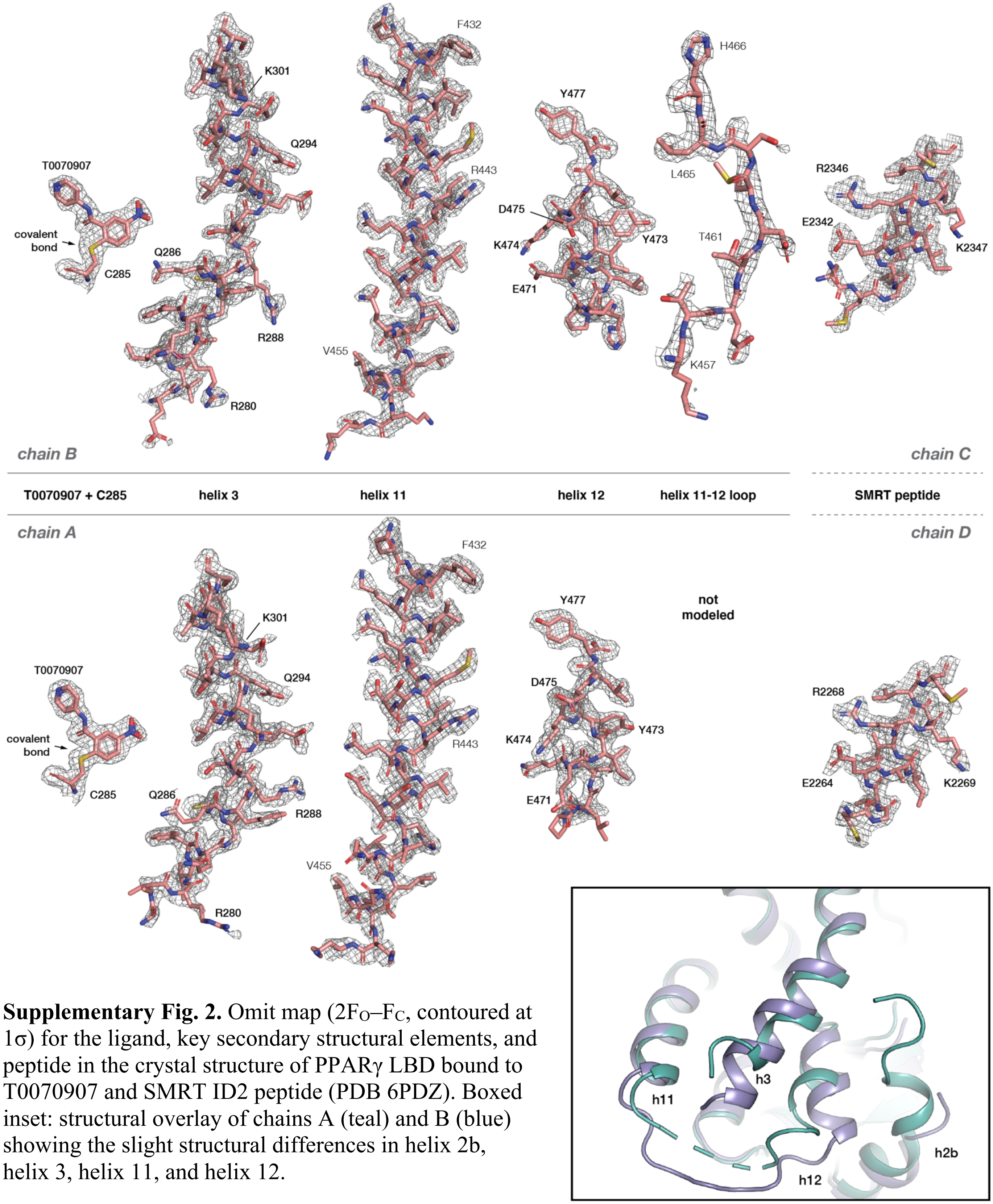

Supplementary Fig. 2. Omit map ($2F_O$–F_C, contoured at 1σ) for the ligand, key secondary structural elements, and peptide in the crystal structure of PPARγ LBD bound to T0070907 and SMRT ID2 peptide (PDB 6PDZ). Boxed inset: structural overlay of chains A (teal) and B (blue) showing the slight structural differences in helix 2b, helix 3, helix 11, and helix 12.

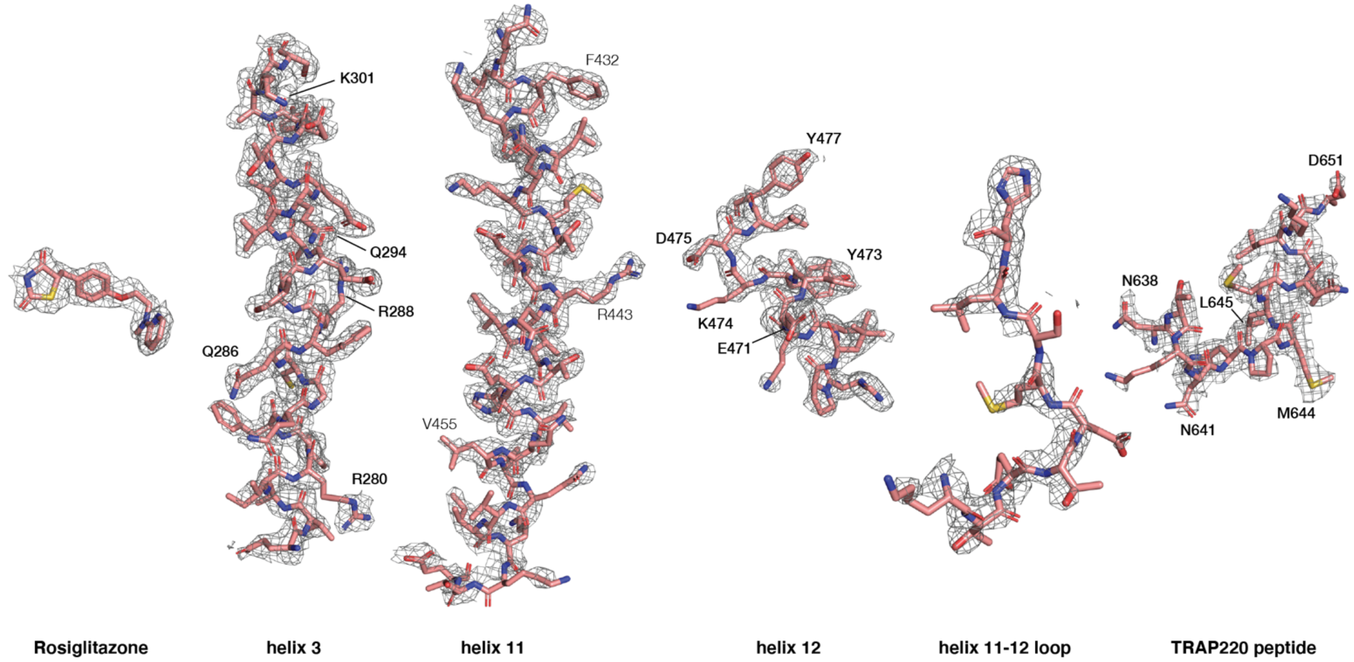

Supplementary Fig. 3. Omit map ($2F_O$–F_C, contoured at 1σ) for the ligand, key secondary structural elements, and peptide in the crystal structure of PPARγ LBD bound to rosiglitazone and TRAP220 ID2 peptide (PDB 6ONJ).

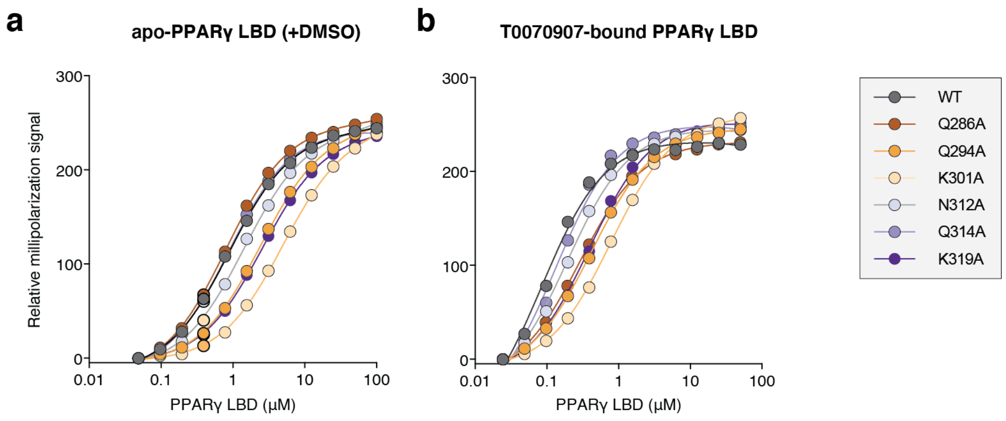

Supplementary Fig. 4. FP assays to determine NCoR ID2 peptide binding affinity. Titration curves (n=3; mean ± s.d.) with (**a**) apo-PPARγ LBD and (**b**) T0070907-bound PPARγ LBD. Source data are provided as a Source Data file.

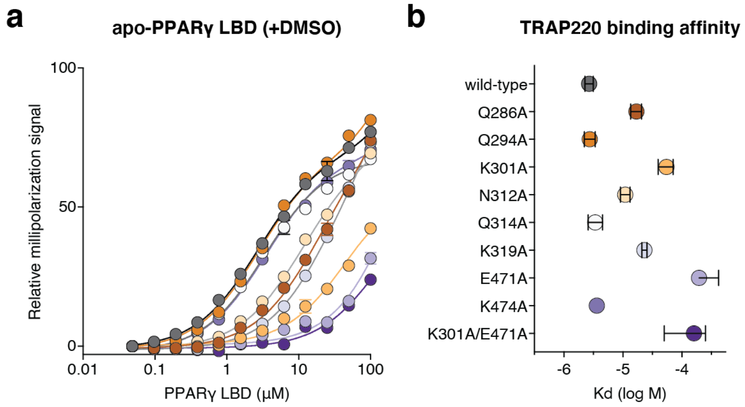

Supplementary Fig. 5. FP assays to determine TRAP220 ID2 peptide binding affinity with apo-PPARγ LBD. (**a**) Titration curves (n=3; mean ± s.d.) with apo-PPARγ LBD. (**b**) Binding affinities and errors from the fitted FP data. Source data are provided as a Source Data file.

,

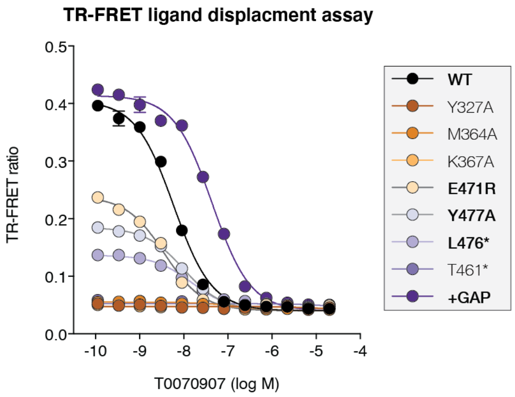

Supplementary Fig. 6. TR-FRET ligand displacement of a fluorescent tracer PPARγ ligand shows that T0070907 binds to most structure-guided mutants (n=3; mean ± s.d.). Several mutants did not show appreciable binding to the fluorescent tracer ligand, including Y327A, M364A, K367A, and T461* (*=stop). Of these, TR-FRET data in **Fig. 6** shows that T0070907 binds to M364A and T461*; for the remaining mutants, liquid chromatography-mass spectrometry data (**Supplementary Table 2**) revealed that T0070907 does not covalently bind to the Y327A and K367A mutants, implicating them in the covalent attachment mechanism. Source data are provided as a Source Data file.

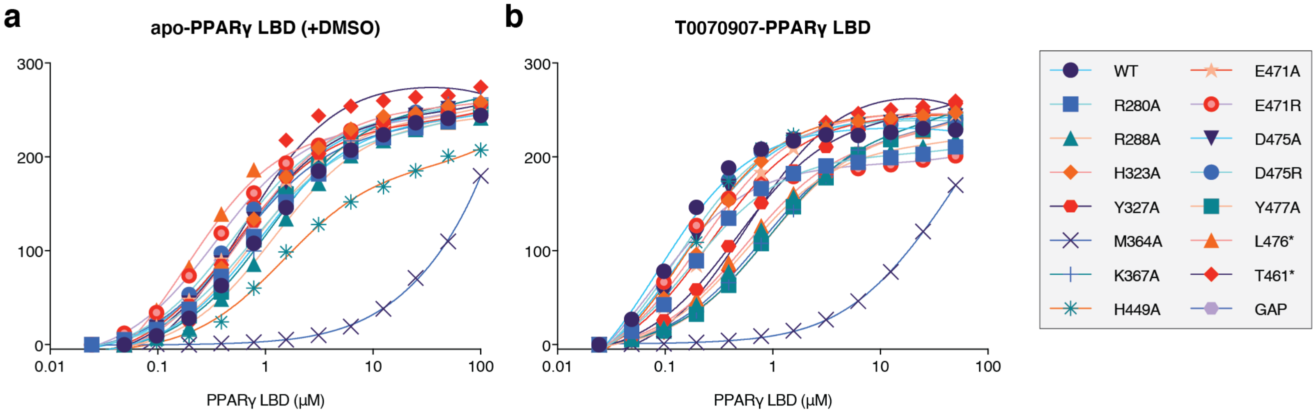

Supplementary Fig. 7. FP assays to determine NCoR ID2 peptide binding affinity with (**a**) apo-PPARγ LBD and (**b**) T0070907-bound PPARγ LBD (n=3; mean ± s.d.). Source data are provided as a Source Data file.

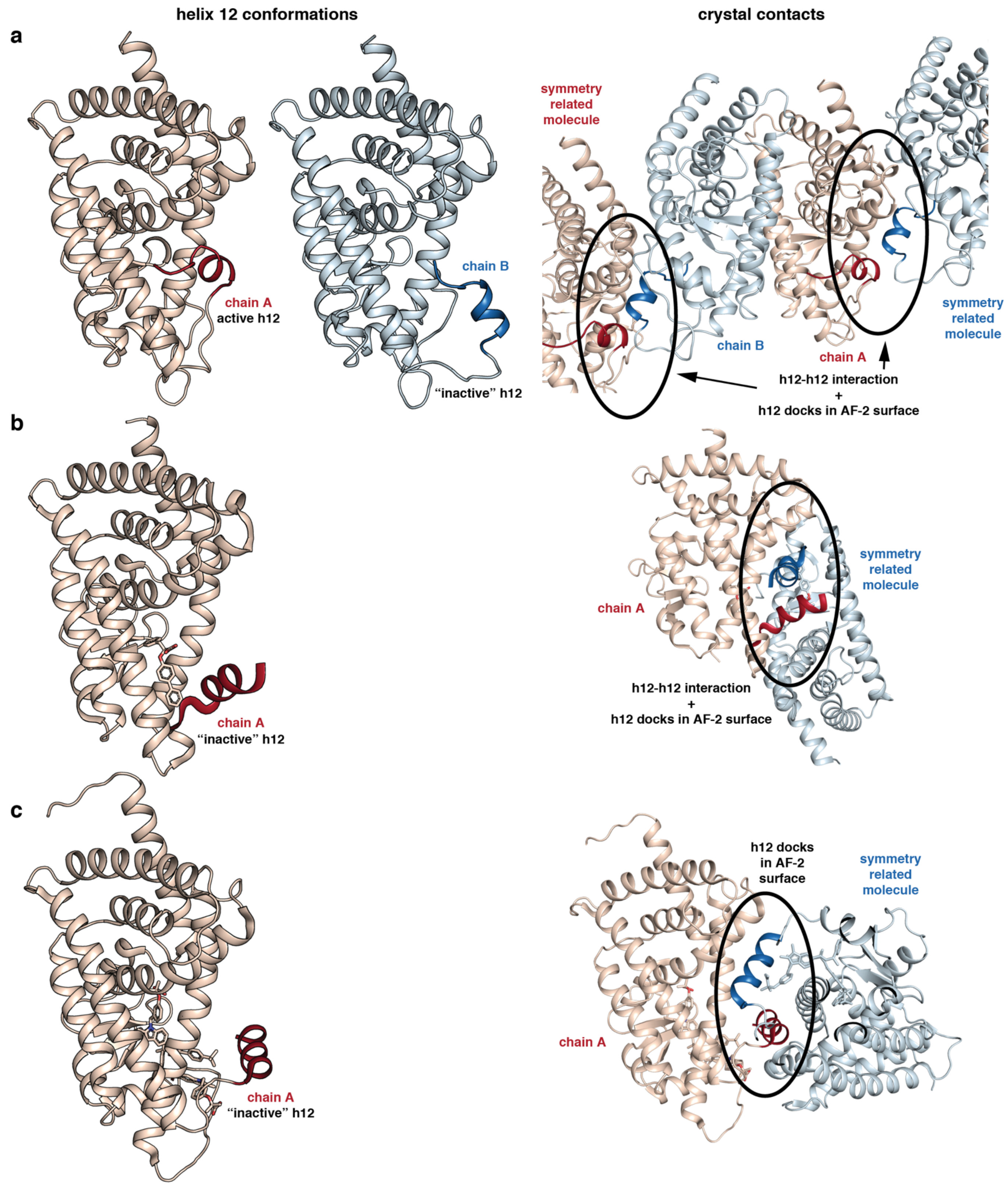

Supplementary Fig. 8. Representative active and non-active helix 12 conformations in PPARγ LBD crystal structures including (**a**) PDB 1PRG (**b**) PDB 4L90, and (**c**) PDB 6C5Q that were solved in the absence of coregulator peptides that are influenced by crystal contacts.

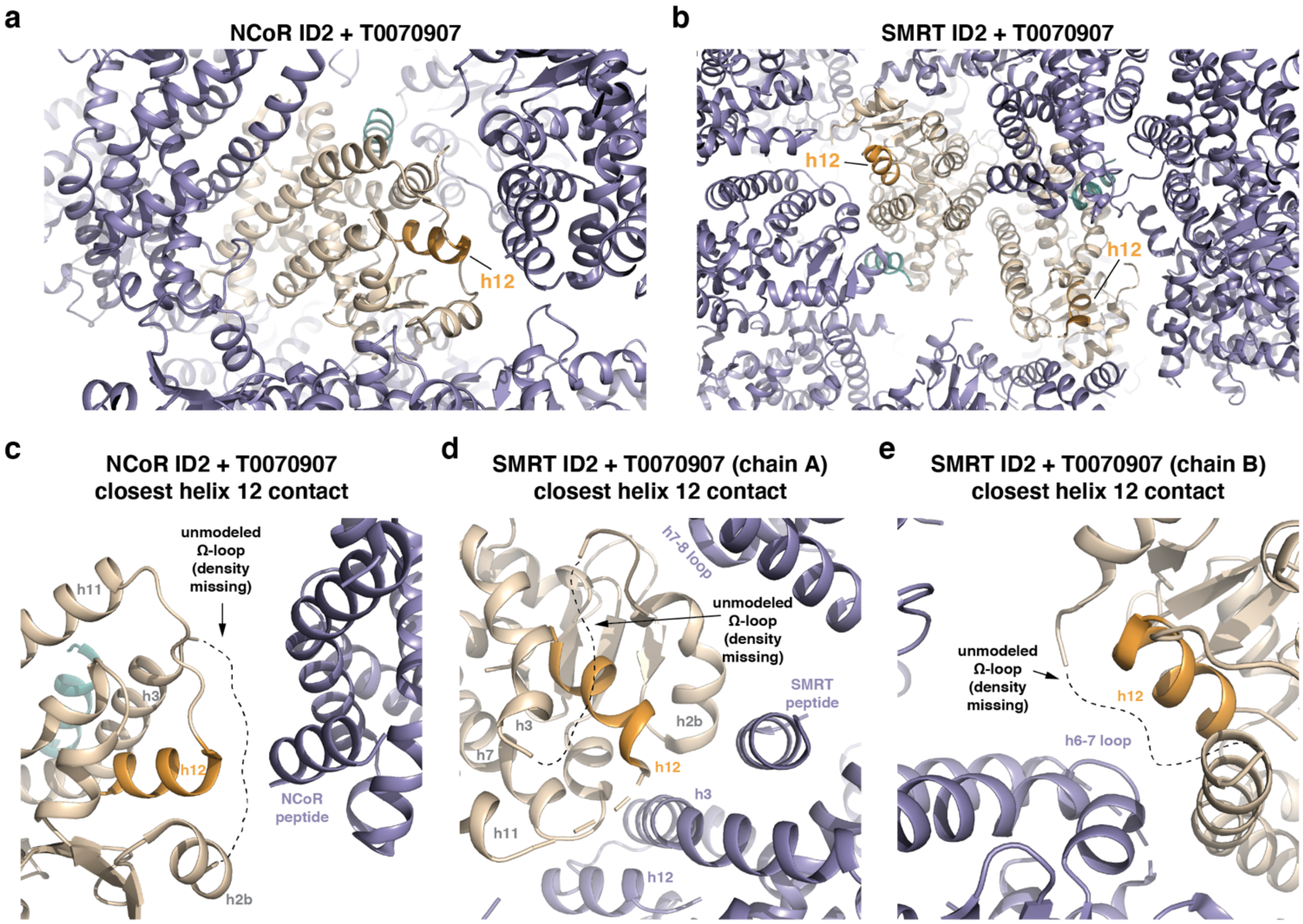

Supplementary Fig. 9. Symmetry related molecules in the crystal structures of PPARγ LBD bound to (**a**) T0070907 and NCoR ID2 peptide (PDB 6ONI) and (**b**) T0070907 and SMRT ID2 peptide (PDB 6PDZ). (**c-e**) Zoomed in view showing the closest helix 12 contact to a neighboring symmetry related molecule. Note that the Ω-loop, which is not visible in the electron density of these structures, is not modeled in—but would form a barrier between the repressive helix 12 conformation and the neighboring symmetry related molecule.

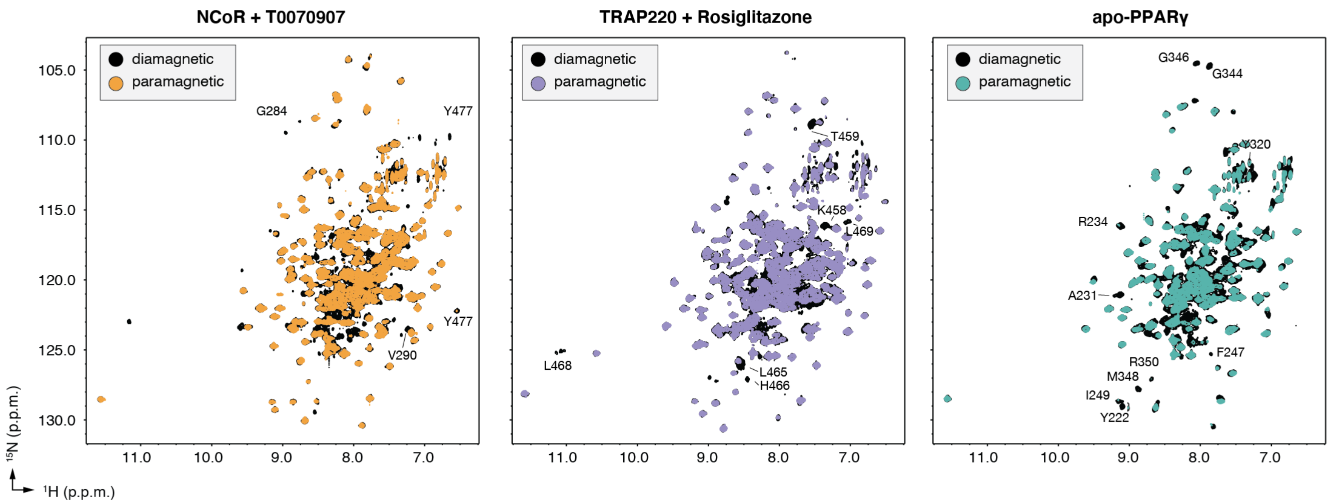

Supplementary Fig. 10. 2D [^{1}H,^{15}N]-TROSY-HSQC NMR PRE data of the repressive, active, and apo-PPARγ LBD conformations collected with a 3-maleimdo-PROXYL spin label show qualitatively similar PRE effects compared to data collected with a MTSL spin label (**Fig. 7**).

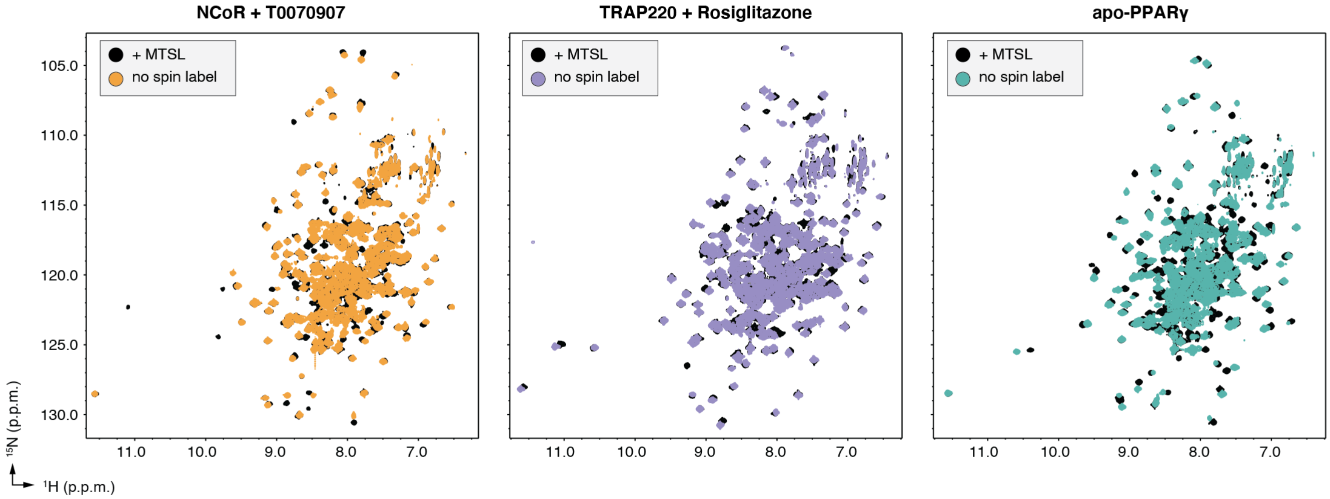

Supplementary Fig. 11. Comparison of 2D [^{1}H,^{15}N]-TROSY-HSQC NMR spectra with and without MTSL spin labels. For the NCoR ID2 peptide + T0070907 overlay, the samples include MTSL-labeled T0070907-bound [K474C]-PPARγ LBD with sodium ascorbate added to reduce the spin label (black) and wild type T0070907-bound PPARγ LBD (orange), both in the presence of 1.5 (orange) or 2 (black) equivalents of NCoR ID2 peptide. For the TRAP220 ID2 peptide + Rosiglitazone overlay, the samples include MTSL-labeled rosiglitazone-bound [K474C/C285S]-PPARγ LBD with sodium ascorbate added to reduce the spin label (black) and wild type rosiglitazone-bound PPARγ LBD, both with 2 equivalents of TRAP220 ID2 peptide. For the apo-PPARγ overlay, samples include MTSL-labeled [K474C/C285S]-PPARγ LBD with sodium ascorbate added to reduce the spin label (black) and [K474C/C285S]-PPARγ LBD (green).

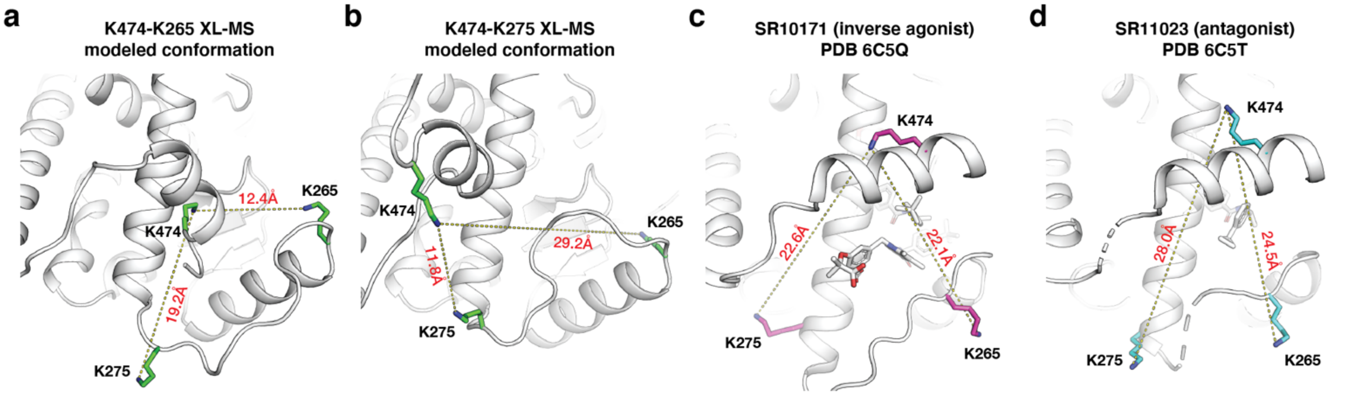

Supplementary Fig. 12. Comparison of distances between three lysine residues in the PPARγ LBD that show increased crosslinks (K474-K265 and K474-K275) in the presence of corepressor peptide. (**a**) Modeled conformation of the PPARγ LBD based on the corepressor peptide increased crosslink between K474-K265 (SI ref [1]). (**b**) Modeled conformation of the PPARγ LBD based on the corepressor peptide increased crosslink between K474-K275 (SI ref [1]). (**c**) SR10171-bound crystal structure of PPARγ LBD solved without a coregulator peptide (PDB 6C5Q). (**d**) SR11023-bound crystal structure of PPARγ LBD solved without a coregulator peptide (PDB 6C5T). In (**c**,**d**), the lysine side chains were modeled using the mutagenesis command in PyMOL since they were not originally modeled during structure determination likely due to weak electron density in these regions.

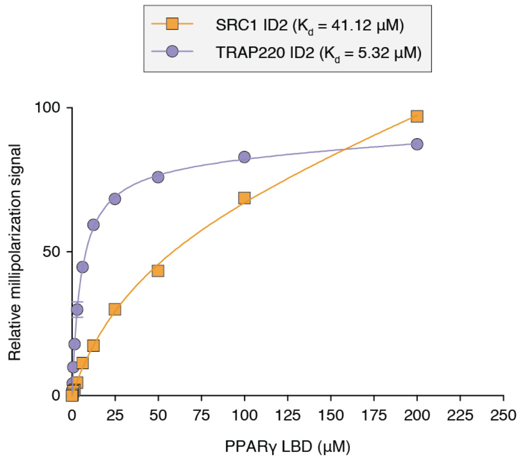

Supplementary Fig. 13. FP assays to determine SRC1 amd TRAP220 ID2 peptide binding affinitys with apo-PPARγ LBD (n=3; mean ± s.d.). Binding affinities from the fitted FP data are listed in the legend. Source data are provided as a Source Data file.

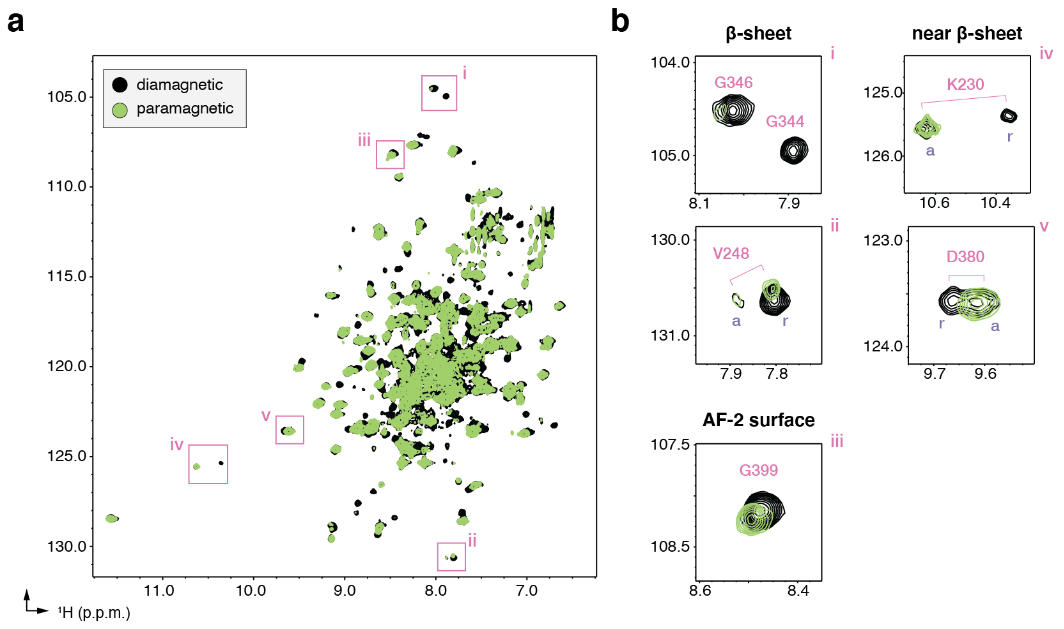

Supplementary Fig. 14. PRE NMR analysis of MTSL-labeled GW9662-bound [K474C]-PPARγ LBD without coregulator peptide. (**a**) 2D [^{1}H,^{15}N]-TROSY-HSQC spectra; pink boxes in (**a**) highlight residues shown the zoomed-in snapshots (**b**).

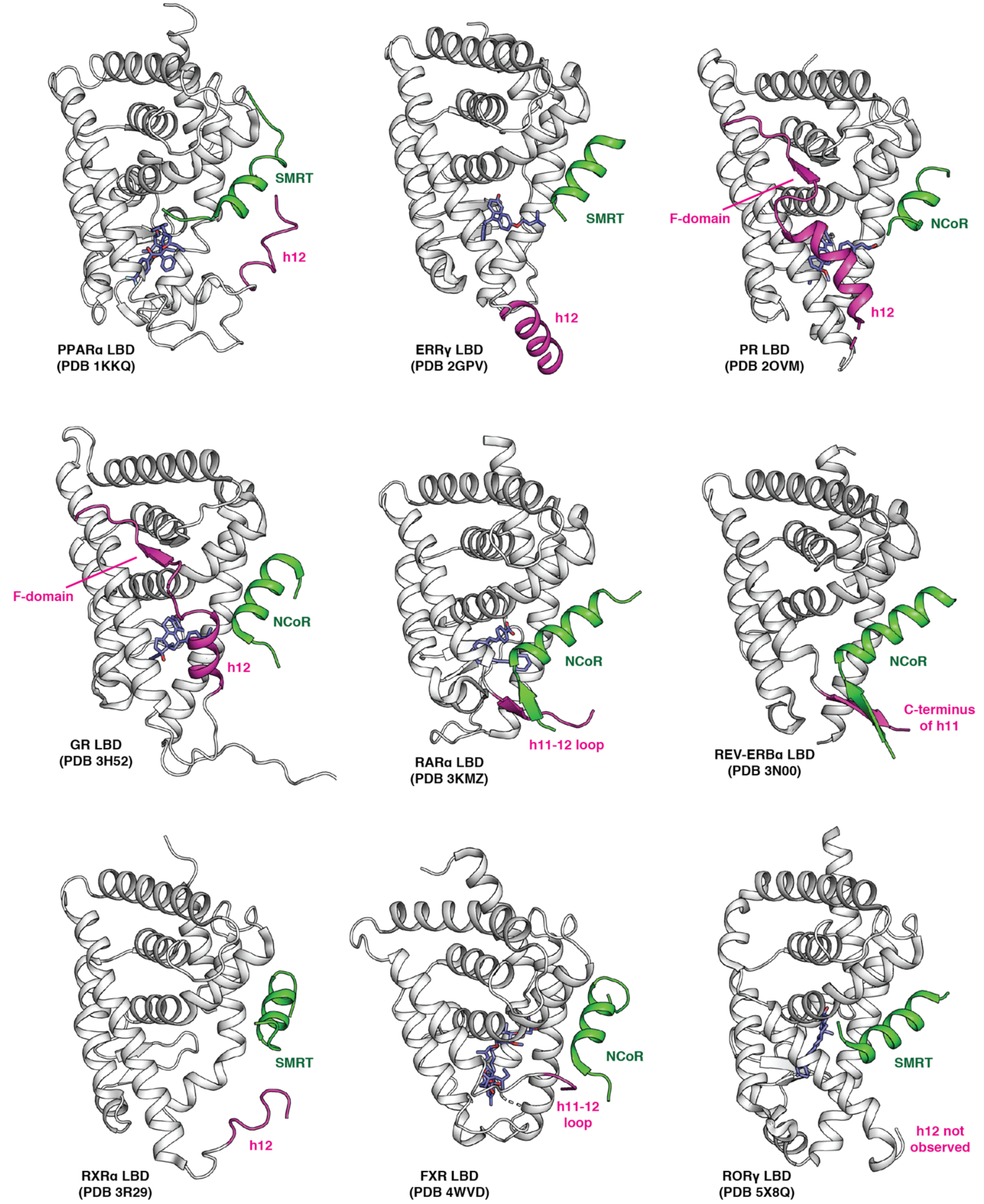

Supplementary Fig. 15. Corepressor-bound NR LBD crystal structures present in the PDB show structurally diverse helix 12 conformations.

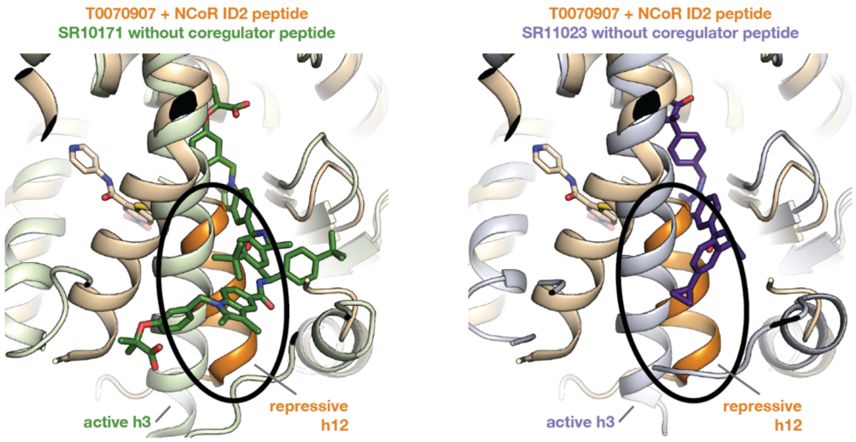

Supplementary Fig. 16. Structural clash of the repressive helix 12 conformation in the PPARγ LBD crystal structure bound to T0070907 and NCoR ID2 peptide (orange) with helix 3 in the active conformation and synthetic ligands bound to the orthsoteric pocket including noncovalent inverse agonist SR10171 (green; PDB 6C5Q) and noncovalent antagonist SR11023 (purple; PDB 6C5T). Ligands are shown as sticks.

Supplementary Table 1. X-ray crystallography data collection and refinement statistics.

	PPARγ LBD bound to Rosiglitazone and TRAP220 ID2 peptide	PPARγ LBD bound to T0070907 and NCoR ID2 peptide	PPARγ LBD bound to T0070907 and SMRT ID2 peptide
**Data collection**			
Space group	C 2 2 2_1	P 4_1 2_1 2	P 2_1 2_1 2_1
Cell dimensions			
a, *b*, *c* (Å)	54.39, 85.57, 121.69	61.88, 61.88, 164.38	59.76, 93.48, 114.61
α, β, γ (°)	90, 90, 90	90, 90, 90	90, 90, 90
Resolution (Å)	36.64-2.30 (2.38-2.30)	34.23-1.80 (1.86-1.80)	36.82-2.10 (2.18-2.10)
R_{sym} or R_{merge}	0.023 (0.157)	0.016 (0.138)	0.031 (0.236)
$I / \sigma I$	16.54 (4.37)	21.08 (3.29)	12.40 (3.00)
Completeness (%)	99.64 (99.76)	99.49 (99.93)	99.60 (99.45)
Redundancy	2.0 (2.0)	2.0 (2.0)	2.0 (2.0)
**Refinement**			
Resolution (Å)	2.30	1.80	2.10
No. reflections	12926	30550	38114
R_{work} / R_{free}	21.8/26.4	22.3/22.6	19.3/23.9
No. atoms			
Protein	2185	2202	4350
Ligand/ion	25	18	36
Water	104	391	532
B-factors			
Protein	29.87	29.39	26.19
Ligand/ion	24.65	24.10	24.61
Water	30.50	30.65	31.74
R.m.s. deviations			
Bond lengths (Å)	0.010	0.007	0.008
Bond angles (°)	1.48	0.88	1.22

*Values in parentheses are for highest-resolution shell.

Supplementary Table 2. ESI-MS data to assess covalent binding to T0070907 to 6xHis-PPARγ LBD.

	Theoretical molecular weight (Da)<br><br>control	Molecular weight from ESI-MS[1] (Da)<br><br>control	Theoretical molecular weight[2] (Da)<br><br>+T0070907	Molecular weight from ESI-MS[1,2] (Da)<br><br>+T0070907	ESI-MS molecular weight difference (Da)<br><br>(control - T0070907)
WT	34107.6	33975	34348.8	34217	242
Y327A	34015.4	33883	34256.6	33833	0
K367A	34050.4	33918	34291.6	33918	0

[1] Molecular weight consistent with a missing N-terminal methionine (149 Da)
[2] Molecular weight of T0070907 without the Cl leaving group is 242.2 Da.

Supplementary Table 3. Primers used for site-directed mutagenesis.

Mutation	Primer sequence
R280A	5'-GAGGTGGCCATCGCTATCTTTCAGGG-3’
C285S	5’-CATCTTTCAGGGCAGCCAGTTTCGCTC-3'
Q286A	5’-CATCTTTCAGGGCTGCGCATTTCGCTCCGTGGAG-3’
R288A	5’-TTTCAGGGCTGCCAGTTTGCCTCCGTGGAG-3’
Q294A	5'-GTGGAGGCTGTGGCAGAGATCACAGAG-3’
K301A	5'-CAGAGTATGCCGCCAGCATTCCTGG-3’
N312A	5'-GTAAATCTTGACTTGGCAGACCAAGTAACTCTC-3’
Q314A	5'-GACTTGAACGACGCAGTAACTCTCC-3’
K319A	5'-GTAACTCTCCTCGCATATGGAGTCC-3’
H323A	5'-CTCAAATATGGAGTCGCCGAGATCATTTACAC-3'
Y327A	5'-CACGAGATCATTGCAACAATGCTGGC-3’
M364A	5'-CTTTTGGTGACTTTGCAGAGCCCAAGTTTG-3’
K367A	5'-CTTTATGGAGCCCGCGTTTGAGTTTGC-3’
H449A	5'-GATTGTCACGGAAGCAGTGCAGCTACTG-3’
T461*	5'-CAAGAAGACGGAGTAAGACATGAGTCTTC-3’
E471A	5'-GCTCCTGCAGGCAATCTACAAGGAC-3’
E471R	5’-CCGCTCCTGCAGAGGATCTACAAGG-3’
K474A	5'-CAGGAGATCTACGCAGACTTGTACTAG-3’
K474C	5'-CAGGAGATCTACTGCGACTTGTACTAG-3’
D475A	5’-GGAGATCTACAAGGCCTTGTACTAACCGG-3’
D475R	5’-CAGGAGATCTACAAGCGCTTGTACTAACCGG-3’
L476*	5'-CTACAAGGACTAATACTAACCGGGC-3’ (pET46-PPARγ-LBD)<br>5’-GAGATCTACAAGGACTAATACTAGACGCGTTAAG-3’ (pCMV-FL-PPARγ)
Y477A	5'-CTACAAGGACTTGGCATAACCGGGCTTC-3’ (pET46-PPARγ-LBD)<br>5’-CTACAAGGACTTGGCATGAGCGCCATGAG-3’ (pCMV-Gal4-PPARγ)<br>5’-CTACAAGGACTTGGCATAGACGCGTTAAG-3' (pCMV-FL-PPARγ)
+GAP	5'-GACTTGTACGGCGCGCCGTAACCGGGCTTC-3’ (pET46-PPARγ-LBD)<br>5’-GGAGCACCATAGACGCGTTAAGCG-3’ (pCMV-FL-PPARγ)

Primers used to generate mutations on the C-terminus required sequence-specific changes (L476*, Y477A, +GAP) due to the different vector sequences.

Supplementary References

1. Zheng J, *et al.* Chemical Crosslinking Mass Spectrometry Reveals the Conformational Landscape of the Activation Helix of PPARgamma; a Model for Ligand-Dependent Antagonism. *Structure* **26**, 1431-1439 e1436 (2018).
