# Supplementary material for: A molecular switch regulating transcriptional repression and activation of PPARγ
Source: Nat Commun. 2020 Feb 19;11:956. doi: 10.1038/s41467-020-14750-x (PMC7031403; doi:10.1038/s41467-020-14750-x)
Supplement: Supplementary file 3 — Description of Additional Supplementary Files [file 41467_2020_14750_MOESM3_ESM.pdf]

Description of Additional Supplementary Files

File Name: Supplementary Movie 1

Description: Structural extrapolation between the active conformation (PDB 6ONJ) and repressive conformation (PDB 6ONI) performed using the morph conformations plug-in within Chimera to illustrate the different conformations of helix 12 (colored magenta) in the active (solvent exposed) and repressive (within the orthosteric ligandbinding pocket) crystal structures.
