# Supplementary material for: A molecular switch regulating transcriptional repression and activation of PPARγ
Source: Nat Commun. 2020 Feb 19;11:956. doi: 10.1038/s41467-020-14750-x (PMC7031403; doi:10.1038/s41467-020-14750-x)
Supplement: Supplementary file 5 — Reporting Summary [file 41467_2020_14750_MOESM5_ESM.pdf]

nature research

Corresponding author(s): Douglas Kojetin

Last updated by author(s): Jan 27, 2020

Reporting Summary

Nature Research wishes to improve the reproducibility of the work that we publish. This form provides structure for consistency and transparency in reporting. For further information on Nature Research policies, see Authors & Referees and the Editorial Policy Checklist.

Statistics

For all statistical analyses, confirm that the following items are present in the figure legend, table legend, main text, or Methods section.

n/a	Confirmed	
☐	☒	The exact sample size (*n*) for each experimental group/condition, given as a discrete number and unit of measurement
☐	☒	A statement on whether measurements were taken from distinct samples or whether the same sample was measured repeatedly
☒	☐	The statistical test(s) used AND whether they are one- or two-sided<br>*Only common tests should be described solely by name; describe more complex techniques in the Methods section.*
☒	☐	A description of all covariates tested
☒	☐	A description of any assumptions or corrections, such as tests of normality and adjustment for multiple comparisons
☐	☒	A full description of the statistical parameters including central tendency (e.g. means) or other basic estimates (e.g. regression coefficient) AND variation (e.g. standard deviation) or associated estimates of uncertainty (e.g. confidence intervals)
☒	☐	For null hypothesis testing, the test statistic (e.g. *F*, *t*, *r*) with confidence intervals, effect sizes, degrees of freedom and *P* value noted<br>*Give P values as exact values whenever suitable.*
☒	☐	For Bayesian analysis, information on the choice of priors and Markov chain Monte Carlo settings
☒	☐	For hierarchical and complex designs, identification of the appropriate level for tests and full reporting of outcomes
☒	☐	Estimates of effect sizes (e.g. Cohen's *d*, Pearson's *r*), indicating how they were calculated

Our web collection on statistics for biologists contains articles on many of the points above.

Software and code

Policy information about availability of computer code

Data collection	Bruker Topspin version 3.2, Gen5 version 2.08
Data analysis	Graphpad Prism verson 8, NMRFx processor version 10.3.49, NMRViewJ version 9.2.0-b24, Mascot, Plink version 2.3.6, Coot version 0.8.9.2, CCP4 version 7.0.078, PHENIX version 1.11.1-2575-000

For manuscripts utilizing custom algorithms or software that are central to the research but not yet described in published literature, software must be made available to editors/reviewers. We strongly encourage code deposition in a community repository (e.g. GitHub). See the Nature Research guidelines for submitting code & software for further information.

Data

Policy information about availability of data

All manuscripts must include a data availability statement. This statement should provide the following information, where applicable:

- Accession codes, unique identifiers, or web links for publicly available datasets
- A list of figures that have associated raw data
- A description of any restrictions on data availability

Crystal structures generated during the current study are available in the Protein Data Bank (PDB) under accession codes 6ONJ (PPARγ LBD bound to rosiglitazone and TRAP220 ID2 peptide), 6ONI (PPARγ LBD bound to T0070907 and NCoR ID2 peptide), and 6PDZ (PPARγ LBD bound to T0070907 and SMRT peptide). NMR chemical shift assignments generated during the current study of PPARγ LBD bound to T0070907 and NCoR ID2 peptide are available in the Biological Magnetic Resonance Data Bank (BMRB) under entry ID 50000. The source data underlying the Figs. 1a–d, 5c–d, 6c–i, 7c, 8b and Supplementary Figs. 4, 5, 6, 7, and 13 are provided in a separate Source Data file. All other datasets generated and/or analyzed during the current study are available from the corresponding author on reasonable request.

Field-specific reporting

Please select the one below that is the best fit for your research. If you are not sure, read the appropriate sections before making your selection.

☒ Life sciences ☐ Behavioural & social sciences ☐ Ecological, evolutionary & environmental sciences

For a reference copy of the document with all sections, see nature.com/documents/nr-reporting-summary-flat.pdf

Life sciences study design

All studies must disclose on these points even when the disclosure is negative.

Sample size	Sample sizes for assays were not determined using any particular methods but were determined based on our experience in running the assays in terms of the number of replicate points needed to sufficiently describe the data
Data exclusions	No data were excluded from the analyses.
Replication	Experiments were typically repeated at least two times.
Randomization	N/A
Blinding	N/A

Reporting for specific materials, systems and methods

We require information from authors about some types of materials, experimental systems and methods used in many studies. Here, indicate whether each material, system or method listed is relevant to your study. If you are not sure if a list item applies to your research, read the appropriate section before selecting a response.

Materials & experimental systems

n/a	Involved in the study	
☐	☐	Antibodies
☐	☐	Eukaryotic cell lines
☒	☐	Palaeontology
☒	☐	Animals and other organisms
☒	☐	Human research participants
☒	☐	Clinical data

Methods

n/a	Involved in the study	
☒	☐	ChIP-seq
☒	☐	Flow cytometry
☒	☐	MRI-based neuroimaging

Antibodies

Antibodies used	LanthaScreen Elite Tb-anti-His Antibody (ThermoFisher #PV5895)
Validation	We used control TR-FRET conditions to determine that the LanthaScreen Elite Tb-anti-His Antibody performs as expected.

Eukaryotic cell lines

Policy information about cell lines

Cell line source(s)	HEK293T (ATCC CRL03216)
Authentication	morphology
Mycoplasma contamination	Cells tested negative for mycoplasma contamination in June 2019.
Commonly misidentified lines (See ICLAC register)	N/A
